# What is important in the surroundings in order to extend the healthy life period? A regional study of 19 older women in a northern part of Norway

**DOI:** 10.3402/ijch.v72i0.21189

**Published:** 2013-08-05

**Authors:** Gunn-Tove Minde, Torill M. Sæterstrand

**Affiliations:** 1 The Health and Social department, Harstad University College, Harstad, Norway; 2 University of Nordland, Bodø, Norway

**Keywords:** elderly women, Sami elderly women, activity, health, participation

## Abstract

**Introduction:**

Participating in a community with other retired individuals to increase life quaøity can be possible for the older persons. Cultural and ethnical background is important for their social identity.

**Objective:**

To identify what the informants think is important in their surroundings in order to extend their healthy life period.

**Study design:**

A structured questionnaire developed by the OCIN network.

**Methods:**

Nineteen elderly women aged 75 years or more were interviewed. This regional survey is a pilot study in Norway. The data were collected during 2 periods, in 2009 and 2010. The data are analyzed using a result scheme prepared by the network OCIN.

**Results:**

Our findings show that this is a group of elderly women that are concerned with promoting their own health. The participants wish to take care of themselves, so they do not become a burden for society and the local authorities.

**Conclusions:**

The findings of this study suggest that participation in the local context is important for promoting health and well-being among elderly in all ethnicities. For the Sami elderly, this is particularly important because meeting equal-minded people helps them maintain their Sami identity. In the Sami culture and among the Sami elderly, it is important to be “strong” and “healthy”. Due to these norms, the elderly Sami women try to live with their illnesses and are less eager to go to the doctor when they are seriously ill.

This article is about health and well-being among elderly women in the northern part of Norway. The informants of this regional survey are people older than 69 years who live within a limited local area. This area is the most populous region of northern Norway with different ethnic groups of elderly – the Sami, the Kven and the ethnic Norwegian. The Sami people are the indigenous people of Norway, the Kven are Finnish people who moved to the coast of Norway during the 18th century. Today, the Kven have a status as a national minority.

This is the first study about elderly health and well-being in Troms and northern part of Nordland in northern Norway. Our research question is: “What do they think is important in their surroundings in order to extend their healthy life period?”

## Cultural context, selection, study design, methods and analysis

The selection of our regional survey is people older than 69 who live within a limited local area of the northern part of Norway. The informants interviewed live in a small town (10,000 inhabitants); 3 regional centres around a town with 35,000 inhabitants; and in sparse rural areas (municipality with 2,500 inhabitants). This area is populated by people with Sami or Finnish heritage including ethnic Norwegians. The Sami area is called the “Markesami area”. This area is characterized by migration from the Swedish side of the border to the coast during the 18th century. The people speak Norwegian, but they have a strong Sami identity.

This regional survey used a structured questionnaire and is part of a bigger European interview survey in 5 countries. The authors of this article have collected these data as part of their research activities. The data were collected in 2009 (1) and 2010 (2). Five participants were recruited through family, friends and neighbors; the rest were recruited through municipal activity centres, retirement organizations and meetings at other social arenas. The data are analyzed using a result scheme prepared by the OCIN-network. Ethnicity is not a question in the survey, but it emerges during the interviews. The questions are in English, but are asked in the Norwegian language.

## Informants: background information

Nine informants live in a small town, 5 live in regional centrese and 5 live in sparsely rural areas. Five of nine informants in a small town have a Finnish or Kven background. Three of 5 informants in the rural areas have a Sami background.

Medium age of informants is roughly 78-and-half years. Seven informants in the small town live with a spouse and 2 live alone. In the regional centres, 3 reside with their spouse, and 2 reside alone. In the rural areas, 2 live with their spouse and 3 reside alone.

In the small town, 8 informants live in their own home or flat, and 1 lives in rented accommodation. In the regional centres, 2 informants live in their own house, and 3 live in rented accommodation. In the rural area, 4 informants live in their own home, and 1 lives in rented accommodation.

Everyone in the survey has some sort of confidant. For 7 informants in the small town, the spouse is their confidant, for 1 informant it is a son or daughter, and for 1 informant the sister is her confidant. In the regional centres, the daughter is the confidant for 4 informants, and for 1 informant the spouse is her confidant. In the rural areas, the spouse is the confidant for 2 informants, and 3 informants have a friend as their confidant.

Concerning education, 6 informants in the small town answer that they have secondary school, 1 informant has elementary school, and 2 informants have higher education. In the regional centres, 4 informants answer that they have elementary school and 1 informant has secondary school education. In the rural areas, 2 informants answer that they have elementary school, while 3 informants have secondary education.

Fifteen informants of the survey feel that their income is sufficient: 8 informants in the small town, 4 informants in the regional centre and 3 in the rural areas. The main source of income is state pension for all informants. Five of the nine informants in the small town have income support pension, while 4 in the regional centres and 1 informant in the rural areas have other sources of income (wage, salary) in addition to pension.

In the small town, 4 consider their health as better than people their own age, and 5 consider their health the same as other people their own age. In the regional centres, 3 feel their health is the same as people their own age, and 2 feel it is worse than people their own age. In rural areas, 3 feel their health is the same as people their own age, 1 informant feels her health is better, and 1 informant feels her health is worse than people their own age.

To the question “How do you carry out the activities of your daily living”, 16 informants in the survey answered that they managed without difficulties, and 3 managed with difficulties (mobility help/wheel chair). Three informants who need help are living in the small town.

## Results

Our findings show that this is a group of elderly women who are concerned with promoting their own health. The surroundings are important to them in order to promote their own health. The findings are divided into 5 topics: social relations, activity, internet, public care, and cultural diversity.

### Social relations

All the informants have children and 11 of the informants have grandchildren. The informants expressed that they did not feel alone because they had good contact with their children and other family members, even if the children lived in different parts of the country. Eight of the informants have contact with their nearest relatives daily. Ten informants mentioned that their nearest relatives helped them with housework. Half of the informants volunteer in helping young and elderly people.

The informants also say they have very good contact with their neighbors, especially those who had moved to more central places and who lived in sheltered housings or apartment buildings. They did not have to go out in order to see others, which is practical for someone who is at risk of slipping and falling on the ice during winter.

One informant living in the regional centers told us she felt lonely. She only had 1 good friend, and it could go weeks between each time they had any contact with each other. Sometimes, they would meet in person, but they basically kept in touch by telephone. The feeling of loneliness is different out in the country side. They are used to not having so many people around. The 3 Sami women, who live out in rural areas, do not feel alone as long as they are able to get out and get around sometimes.

It seems that approaching the loneliness topic can be quite emotional and difficult to talk about. To tell others that they feel lonely is a big shame for the informants.

### Activity

The informants are keen to get out and participate in various social activities almost every week, especially in the day light seasons (February–October). They feel that it is important to keep active and to be out in the fresh air. Single women felt isolated when the roads were not cleared enough during winter time, because it made them reluctant to drive their cars other places than on the main roads. An older woman in the rural area said that she did not dare drive from her home to the activity center due to the municipality's road quality. One single women living in a small town complained about the poor public transportation. Two of the Sami women depend on their husbands or children in order to get out. They had to get a lift or be offered a ride to go see neighbors, visit the regional center to go shopping, or attend Læstadianian gatherings (faith within Christianity) and other communal meetings taking place in the region. The third Sami woman had gotten her own car in order to be independent of her husband.

### Radio, TV, newspapers and the Internet

Our findings also show that all of the elderly in this study have access to TV and radio. They want to stay updated on what is happening in the local community and society. Six informants, 5 in a small town and 1 in the rural area, have access to the Internet.

### Public care

Few of the informants receive home care from the public care system. Most of the informants receive instead assisted living from the municipality. Assisted living is housework provided by paid house-keepers.

### Cultural diversity

There was cultural diversity at the different activity centres we visited. The informants of Kven/Finnish background blend into the townscape without their ethnicity being relevant. It is still considered disgraceful to talk openly about their ethnic background. Even their children do not know that they have Sami or Kven family background. Despite this, their cultural and ethnical background is very important to them. It is a part of their social identity. Yet, they chose to adapt to the small town's norms and values by hiding their own ethnicity.

## Discussion

Our study shows that our informants have different needs and wishes for their golden years, as other studies have revealed (3,4). Our selection is a convenient sample, and the informants were recruited from different contexts; that is small town, regional centres, and rural areas. Our findings show that the local context is important for the elderly and they have the knowledge and are aware of how they can stay healthy when they reach higher ages.

Society's attitude and political currents of how they see themselves as elderly affects them in many different ways. It does not only affect their identity and self-esteem, but also their inclination to seek help. Our findings may indicate that they still are silent when meeting the social-help network. We found that the elderly declared an existential loneliness and decided to settle on being alone and an outsider of society. There are cultural differences. Older women from Sami communities play a meaningful role in relation to up-bringing, care-taking and knowledge construction in the Sami community.

Furthermore, our findings show that the older person is different and no longer lives up to the picture painted of the passive patient who put their lives in the hands of the caretakers. Several of our informants experienced better health and a healthier life by participating in the activity centres and different organizations, such as retirement associations and senior councils. The participation in the monthly meetings in retirement associations or other associations was the main source of contact with others. If they had not gone to these meetings, they would be sitting in retirement homes staring at the wall as 1 informant stated. One informant, a widower, was highly active in 11 associations which served her need for social interaction. According to Alm Andreassen (5) the patients work to re-orientate themselves, their life project, and their understanding of themselves, by defining their problem and mapping alternative solutions. This happens to some who have experienced being retired because of their health problems. It may seem that the work or the orientation can be compared to what Antonovsky (6) calls the salutogenetic work, because they are focusing on the resources of each other and see the elderly in their context. The elderly were more content with their lives by participating in the social community. Thus, you can say that by participating in a community with other retired individuals, increased life quality is possible for the older person. The informants are also concerned with participating in different associations and organizations, some mostly because of the social aspect. As an older person you are no longer involved in a working life and are in need of other forms of confirmation. The need for participation and being able to affect ones surroundings seems to give people better self-esteem and feeling of mastery, as well as improving their mental health (1,7,8). Feeling cultural belonging and connection as Antonovsky (6) defines, is probably something many people are looking and longing for today, and is a quality the informants in this study hold.

Activity seems to be the keyword. The fact that our older informants are able to get out of the house to participate in activities is important, and society needs to arrange for this to happen. According to Kleinmann's (9) theory on health care systems, it is the family sector that mainly takes care of the relative's health. Studies in the northern areas show that this in particular is the woman's domain and that is followed up by the younger generation (10,11). In our study, everyone has a confidant even if their spouse is deceased. It is their spouse, son, daughter, or another close relative. At the same time, they emphasize the importance of their family's role in their ability to get out and about and participate in different activities. There is a difference between the north and south of Norway: the northern part of Norway traditionally has had many ethnic groups live together and they have had a close relationship. We can see that in the emphasis of differences between individualism and collectivism (2). Our findings support this difference. It is a wish and an expectation of the family and extended social network to be there for them when they need to get to the store, to the city, etc. When they become dependent on nursing, they prefer to become that to be a public responsibility.

The study shows that the 3 older Sami women living in rural areas are active at older ages. They participate in the religious congregation almost every Sunday where they meet their equals – in local language, culture and religion. Even when they live with physical suffering and diseases, they do not see themselves as old, as long as they can maintain the roles they have always had (12).

Five women from the small town, who have a Sami or Kven family background, say that their cultural and ethnical background is very important to them. Yet, they chose to adapt to the small town's norms and values by hiding who they are ethnically. There is an opinion in the northern part of Norway that the Sami are primary a rural people, and that the Kven people assimilate in the majority culture both in the rural areas and in the small towns. But our study shows that the ethnic people have also moved to small towns. Our research shows that both the Sami and the Kven people are acculturated into the Norwegian majority culture. But the ancient identity is part of their social identity; that is a mix of social identities which include the Sami or Kven identity, or both. In studies of the elderly in northern Scandinavia, findings show that older people are strongly attached to the surroundings they are a part of; where they can maintain the role they have (13–15). Age is no obstacle in order to be included, especially in the rural areas as long as they are mentally well. This especially concerns the older Sami women.

Access to the internet is increasing among the elderly. Through the internet, they can share experiences, and at the same time get in contact with others, such as grandchildren and/or great-grandchildren. This helps to maintain contact with the younger generation, and it keeps the elderly updated on what kind of challenges kids and young people have.

## Method criticism/limitations

Fourteen informants are recruited through municipal activity centres, retirement organizations, and meetings at other social arenas. The process may have led to an uneven selection with many well-functioning elderly, as they were already participating in the local communities. Choosing elderly informants in the small town based on information given by retirement organizations and senior centres may have resulted in us finding elderly with different cultural backgrounds or elderly who hide their Sami and Finnish background. Those who chose a more segregated existence in order to preserve their ethnic background are present in the retirement organizations or senior centres.

OCIN (Older people in Europe) is a network for participants from 5 European countries, Finland, Norway, England, Czech Republic, and Poland. The goal is to collaborate doing research on active ageing in Europe. The data was collected during 2 periods in 2009 and 2010. The data is analyzed using a result scheme prepared by the network OCIN (16).

The researchers’ viewpoint is important. With one of us being ethnic Norwegian and the other being Sami, we believe we have both an insider and outsider perspective. If both were ethnic Norwegian, the danger is that this will affect what respondents want to talk about in an interview. Participants may wish to appear to be grateful and polite in the face of ethnic Norwegian and therefore they will not share their special needs. But with one of the researchers being Sami, we believe we have limited this source of error.

## Conclusions

The findings of this study suggest that participation and activity in the local context is important for promoting health and well-being among the target group. The findings show that the informants are a group of elderly that are concerned with promoting their own health. The elderly women want to take care of themselves so they do not become a burden for society. Our studies also show cultural differences. The elderly Sami women in the target group are active at older ages, especially in arenas where they meet their equals – in language, culture and religion. For the Sami women, this is especially important because meeting equal minded people helps them maintain their Sami identity in sickness and old age. This is important for the feeling of well-being because elderly Sami women are less likely to seek the doctor when they are sick (14).
